# Shift of dietary carbohydrate source from milk to various solid feeds reshapes the rumen and fecal microbiome in calves

**DOI:** 10.1038/s41598-022-16052-2

**Published:** 2022-07-20

**Authors:** Thomas Hartinger, Cátia Pacífico, Gregor Poier, Georg Terler, Fenja Klevenhusen, Qendrim Zebeli

**Affiliations:** 1 Department for Farm Animals and Veterinary Public Health, Institute of Animal Nutrition and Functional Plant Compounds, University of Veterinary Medicine Vienna, Vienna, Austria; 2 Biome Diagnostics GmbH, Vienna, Austria; 3 Institute of Livestock Research, Agricultural Research and Education Centre Raumberg-Gumpenstein, Irdning-Donnersbachtal, Austria; 4 Department Safety in the Food Chain, German Federal Institute for Risk Assessment, Berlin, Germany

**Keywords:** Bacteria, Microbial communities, Environmental microbiology, Microbiology techniques

## Abstract

The transition from milk to solid diets drastically impacts the gut microbiome of calves. We explored the microbial communities of ruminal fluid and feces of Holstein calves when fed milk on d 7 of life, and when fed solid feeds based on either medium- or high-quality hay with or without concentrate inclusion (70% in fresh matter) on d 91. Ruminal fluid and feces had distinct microbial compositions already on d 7, showing that niche specialization in early-life gut is rather diet-independent. Changes between d 7 and d 91 were accompanied by a general increase in microbial diversity. Solid diets differed largely in their carbohydrate composition, being reflected in major changes on d 91, whereby concentrate inclusion was the main driver for differences among groups and strongly decreased microbial diversity in both matrices. Fecal enterotyping revealed two clusters: concentrate-supplemented animals had an enterotype prevalent in *Prevotella*, *Succinivibrio* and *Anaerovibrio,* whereas the enterotype of animals without concentrate was dominated by fibrolytic *Ruminococcaceae*. Hay quality also affected microbial composition and, compared to medium-quality, high-quality hay reduced alpha-diversity metrics. Concluding, our study revealed that concentrate inclusion, more than hay quality, dictates the establishment of niche-specific, microbial communities in the rumen and feces of calves.

## Introduction

During early life, calves are functionally monogastrics (pre-ruminants) and digestion takes place mainly in the abomasum and intestine. The intake of solid feed stimulates the development of the forestomach system and the calf becomes a fully mature ruminant whose digestion is dominated by microbial fermentation in the rumen^1^. The establishment of microbial fermentation constitutes an important physiological event in the calf’s life, meaning the establishment of site-specific microbial niches and structurally as well as functionally diverse microbial communities^2^.

Although it is commonly known that the uptake of solid feed promotes the development of the reticulo-rumen and its associated microbiota^1,3,4^, influences of specific feeding regimes on the intestinal microbiota in calves are scarcely understood. Recent work by Lourenco et al.^5^ demonstrated distinct bacterial compositions in the rumen and feces of 92 d old suckling beef calves supplemented or not with concentrate, showing that concentrate inclusion decreased bacterial genera associated with fiber degradation in the rumen. Likewise, Beharka et al.^6^ earlier proposed that forage diets rich in structural carbohydrates can be beneficial for shaping the rumen environment in calves as evidenced by a higher presence of cellulolytic bacteria and persistence of a physiological rumen pH. However, it is highly conceivable that not only the forage proportion but also the forage quality, i.e. contrasting chemical compositions and digestibility, will affect the development of the calves’ gut ecosystem. In adult dairy cows, different hay qualities were shown to shift the microbial communities at all taxonomic levels in the rumen^7^, such as an increase in *Prevotella*, *Ruminococcus* and *Pseudobutyrivibrio* with feeding hay rich in water-soluble carbohydrates. Likewise, feeding fiber-rich hay with 40% concentrate led to a higher abundance of *Ruminobacter* and RFN20, and a decrease in *Selenomonas* in the free rumen fluid when compared with feeding hay rich in water-soluble carbohydrates with the same amount of concentrate^7^.

In calves, studies on the impact of including concentrate on the gut microbiota are rare and with regards to different forage qualities, alone or in combination with concentrate, yet lacking. However, recent findings from our group indeed demonstrate distinct gut fermentation patterns of Holstein calves switched from milk feeding to solid feed differing in the quality of hay (i.e., high-(HQH) or medium-(MQH)), with (+ C) or without concentrate supplementation^8^. This difference in hay quality and concentrate inclusion resulted in major changes in the carbohydrate composition, especially starch, water-soluble carbohydrates, and fiber, and this also affected calves’ feed intake and growth performance^9^. Knowledge about the roles of both concentrate inclusion and forage quality on the gut microbiota in calves will therefore help to better understand these observations and possibly explain the general implications of varying carbohydrate sources in the gut of rearing calves, which is still largely an open research question.

Consequently, the aim of the present work was first to investigate the changes in the ruminal and fecal microbiota from pre-ruminant to weaned calves, especially as a function of concentrate inclusion and distinct hay qualities. Additionally, we also aimed to clarify whether the changes observed in ruminal and fecal fermentation in response to different solid diets can be attributed to specific changes in the microbiota composition. Therefore, ruminal fluid and feces collected from calves on d 7 and d 91 were analyzed for their microbial community composition, i.e. when animals consumed mainly acidified milk or one of the four solid diets (HQH, MQH, HQH + C, MQH + C), respectively. We hypothesized that introduction of solid feed would lead to a reshaping of the gut microbiota that is driven by both dietary factors, either towards fibrolytic-associated microbes in the gut of purely hay-fed calves or starch degraders in animals fed 70% concentrate.

## Results

### Sample collection and sequencing results

Due to health issues, two animals (one female of the MQH group and one male of the MQH + C group) had to be removed from the experiment and were not included in data analysis. A total of 73 ruminal fluid and 74 fecal samples were included in this analysis. Bacterial and archaeal amplicons were sequenced, yielding a total of 12,913,206 merged reads. After quality filtering, a total of 12,895,338 high-quality reads remained in analysis, representing an average of 87,723.39 ± 35,938.21 reads per sample. The minimum read count per sample was 24,530 reads, while the maximum was 203,232. After denoising and taxonomic assignment, the dataset consisted of a total of 7,696 features belonging to bacteria and 40 belonging to archaea.

### General characteristics of microbial communities in ruminal fluid and feces

Ruminal fluid samples had an average of 98,546.26 ± 34,481.25 reads, while fecal samples had overall lower read counts (77,046.77 ± 34,318.43). Total read counts and % of total read counts at the phylum, family and genus level are given in Supplementary Table 1. Firmicutes, followed by Bacteroidetes and Proteobacteria were the most abundant phyla, accounting to as much as 94.5% and 97.1% of the microbial communities in ruminal fluid and feces, respectively. No Armatimonadetes, Deinococcus-Thermus, Fusobacteria, Synergistetes and Thaumarchaeota were found in feces, as opposed to ruminal fluid. Deferribacteres was only observed in feces. At the genus level, ruminal fluid and feces were largely divergent (Supplementary Fig. 1). The most abundant genera in ruminal fluid were *Prevotella* 1, *Bacteroides*, *Actinomyces*, *Succinivibrionaceae* UCG-001, *Veillonella*, *Prevotella* 7, *Lachnoclostridium*, *Gallibacterium*, *Sharpea* and *Akkermansia*. In feces, *Bacteroides* was the most abundant genus, followed by *Faecalibacterium*, *Bifidobacterium*, *Ruminococcaceae* UCG-005, *Escherichia-Shigella*, *Ruminococcaceae* UCG-010, *Butyricicoccus*, *Prevotella* 9, *Rikenellaceae* RC9 gut group and *Veillonella*.

### Microbial compositions in ruminal fluid and feces of calves fed acidified milk

A total of 300 and 150 microbial genera were found in both ruminal fluid and feces on d 7, respectively (Supplementary Table 2). The top 10 most abundant genera found in ruminal fluid were *Bacteroides*, *Actinomyces*, *Veillonella*, *Gallibacterium*, *Lachnoclostridium*, *Akkermansia*, *Alloprevotella*, *Streptococcus*, *Prevotella* 1 and *Bifidobacterium*. *Bacteroides*, *Bifidobacterium, Veillonella* and *Gallibacterium* were also amongst the top 10 microbial genera found in feces, together with *Faecalibacterium*, *Escherichia*-*Shigella*, *Butyricicoccus*, [*Ruminococcus*] *gnavus* group, *Collinsella* and *Lactobacillus*. From the 150 microbial genera found in feces, 133 were also found in ruminal fluid. *Sellimonas*, *Muribaculaceae* uncultured bacterium, *Negativibacillus*, CAG-56, [*Eubacterium*] *hallii* group, *Coprococcus* 3, *Peptoclostridium*, *Erysipelotrichaceae* UCG-003, *Marvinbryantia*, *Bacteroidales* RF16 group uncultured *Porphyromonadaceae* bacterium, *Carnobacterium*, *Pseudoramibacter*, *Caproiciproducens*, *Fusicatenibacter*, *Intestinibacter*, *Ruminiclostridium* 6 and uncultured *Erysipelotrichaceae* bacterium were exclusively found in feces. Sex did not play a role in the microbial community structure at d 7 (*P* > 0.90). However, an effect of individual variability and matrix was clearly observed for both Aitchison and Bray–Curtis distance matrices (*P* < 0.05).

### Gut microbial community changes from early life to post-weaning

**Figure 1:**
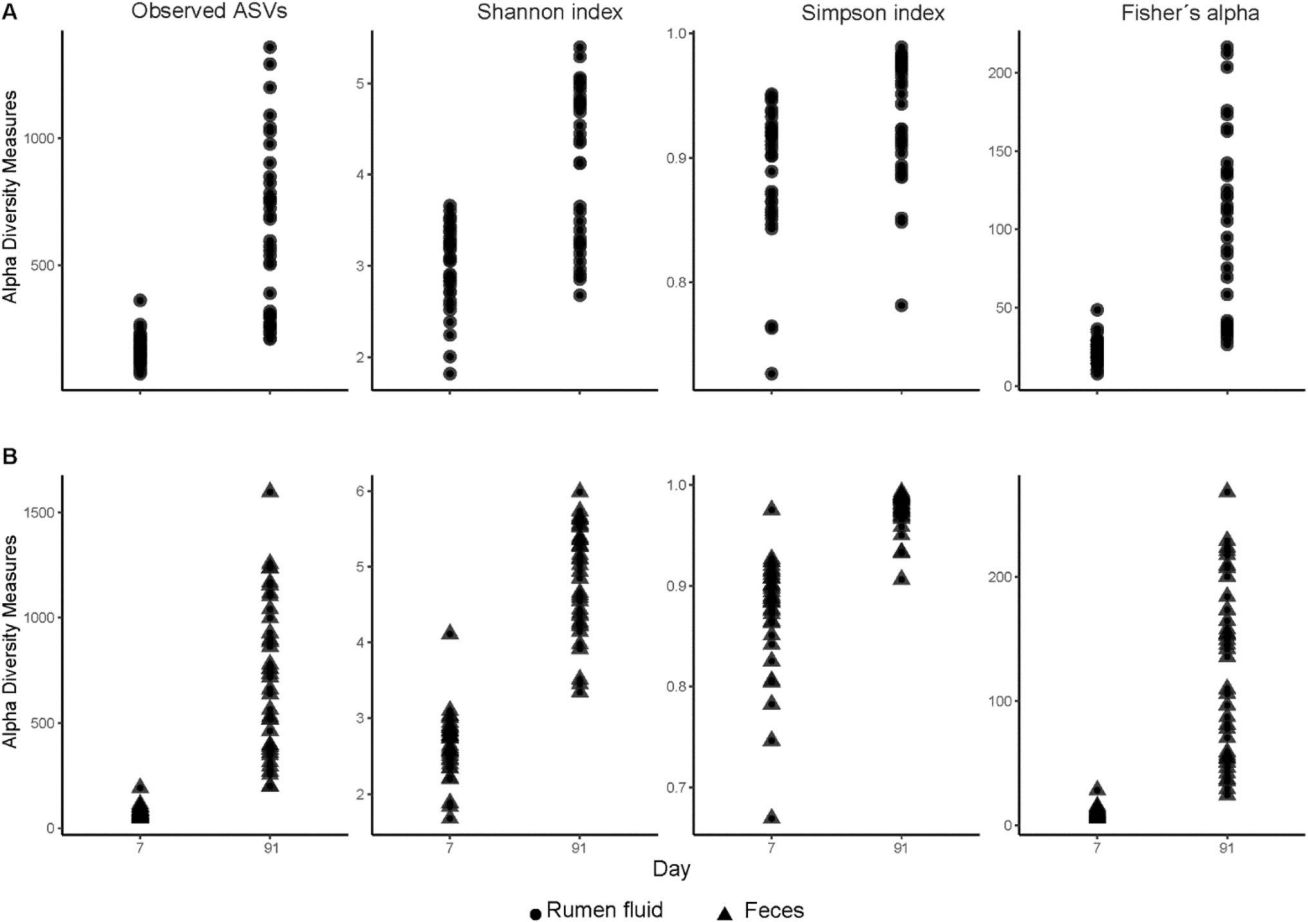
Comparison of alpha-diversity estimates between d 7 and d 91 in ruminal fluid (**A**) and feces (**B**).

The number of observed amplicon sequence variants (ASVs) as well as the determined microbial diversity measures, i.e. Shannon, Simpson and Fisher´s alpha diversity index, were significantly different between d 7 and d 91 in both rumen and feces (*P* < 0.01, Fig. 1A,B). On d 7, a significantly higher number of ASVs was found in rumen samples (185 ± 42), when compared with feces (80 ± 42; *P* < 0.01). However, at d 91, differences between matrices were no longer significant (*P* = 0.365). Shannon and Simpson indexes were not impacted by matrix (*P* > 0.10), but increased between d 7 and d 91 (*P* < 0.01). No significant impact of animal or sex was found for Aitchison and Bray–Curtis distance matrices. Day (*P* < 0.01), followed by matrix (*P* < 0.01), were the strongest factors influencing microbial community structure. When considering changes at the level of the microbial composition, 116 and 72 genera were found to significantly change between d 7 and d 91 in ruminal fluid and feces, respectively (> 2.0 coefficient < − 2.0, Q < 0.05, Supplementary Table 3).

The top 10 genera that most increased in ruminal fluid between d 7 and d 91 were *Succiniclasticum*, *Acetitomaculum*, *Selenomonas* 1, *Prevotellaceae* YAB2003 group, *Prevotellaceae* UCG-001, *Olsenella*, *Lachnospiraceae* NK3A20 group, *Muribaculaceae* uncultured rumen bacterium, *Pseudobutyrivibrio* and *Methanobrevibacter* (coefficient > 4.06, Q < 0.01). *Bacteroides*, *Actinomyces*, *Gallibacterium*, *Lachnoclostridium*, *Veillonella*, *Phascolarctobacterium*, *Bibersteinia*, *Parabacteroides*, *Tyzzerella* and *Escherichia*-*Shigella* (coefficient < − 5.57, Q < 0.01) were amongst the genera that most declined between d 7 and d 91 in ruminal fluid. *Ruminococcaceae* UCG-005, *Rikenellaceae* RC9 gut group, *Eubacterium coprostanoligenes* group, *Muribaculaceae* uncultured bacterium, *Christensenellaceae* R-7 group, *Ruminococcaceae* UCG-010*, Lachnospiraceae* UCG-010, *Alloprevotella*, *Peptococcaceae* uncultured and *Clostridiales* vadinBB60 group uncultured bacterium were amongst the microbial genera that most increased from d 7 to d 91 (coefficient > 3.56, Q < 0.01), while *Ruminococcus gnavus* group, *Veillonella*, *Collinsella*, *Bifidobacterium*, *Actinomyces*, *Gallibacterium*, *Lactobacillus*, *Escherichia*-*Shigella*, *Butyricicoccus* and *Faecalibacterium* were amongst those that mostly decreased in feces (coefficient < − 5.59, Q < 0.01).

### Correlations between microbiota composition, pH and SCFA in ruminal fluid and feces

Spearman correlations between microbiota and fermentation parameters in ruminal fluid and feces on d 91 were considered as strong when r > 0.70 and < − 0.70. Therefore, investigation whether bacterial genera that were predominant in one treatment were correlated with certain microbial metabolites or pH was facilitated. Besides, a correlation network was built for ruminal fluid (Supplementary Fig. 2).

In the ruminal fluid, the strongest negative correlations were found for *Actinomyces, Escherichia-Shigella, Bacteroides, Eggerthella* and *Streptococcus* (r < − 0.80). *Bacteroides, Actinomyces, Eggerthella, Escherichia-Shigella* were found to be negatively correlated with total SCFA, propionic, acetic, butyric, isobutyric and isovaleric acids, while *Streptococcus* was negatively correlated with total SCFA, propionic, acetic, butyric and valeric acids (r < − 0.70). *Methanobrevibacter* was highly associated with the production of isovaleric and isobutyric acids (r = 0.84 and 0.83, respectively). *Oribacterium* was highly correlated with butyric, acetic, isobutyric, isovaleric acid and total SCFA (r > 0.80). *Succiniclastum*, *Selenomonas* 1 and *Prevotellaceae* YAB2003 group were positively correlated with total SCFA, propionic, valeric, acetic and butyric acids. Family XII AD3011 group was found to be highly correlated with isobutyric acid, isovaleric acid and butyric acid (r > 0.80).

In feces, strong correlations were only found for valeric acid (r < − 0.70 or r > 0.70). *Ruminococcus gnavus* group and *Actinomyces* were found to be negatively associated with this SCFA, while 34 other genera were found to be positively associated (r > 0.70).

*Lachnospiraceae* UCG-010*, Acetitomaculum, Erysipelotrichaceae* UCG-004*,* Family XIII AD3011 group*, Eubacterium coprostanoligenes* group*, Oscillibacter, Methanosphaera, Breznakia, Marvinbryantia* and *Roseburia* were positively correlated with valeric acid (r > 0.75).

### Microbial diversity in ruminal fluid and feces in response to different solid diets

**Figure 2:**
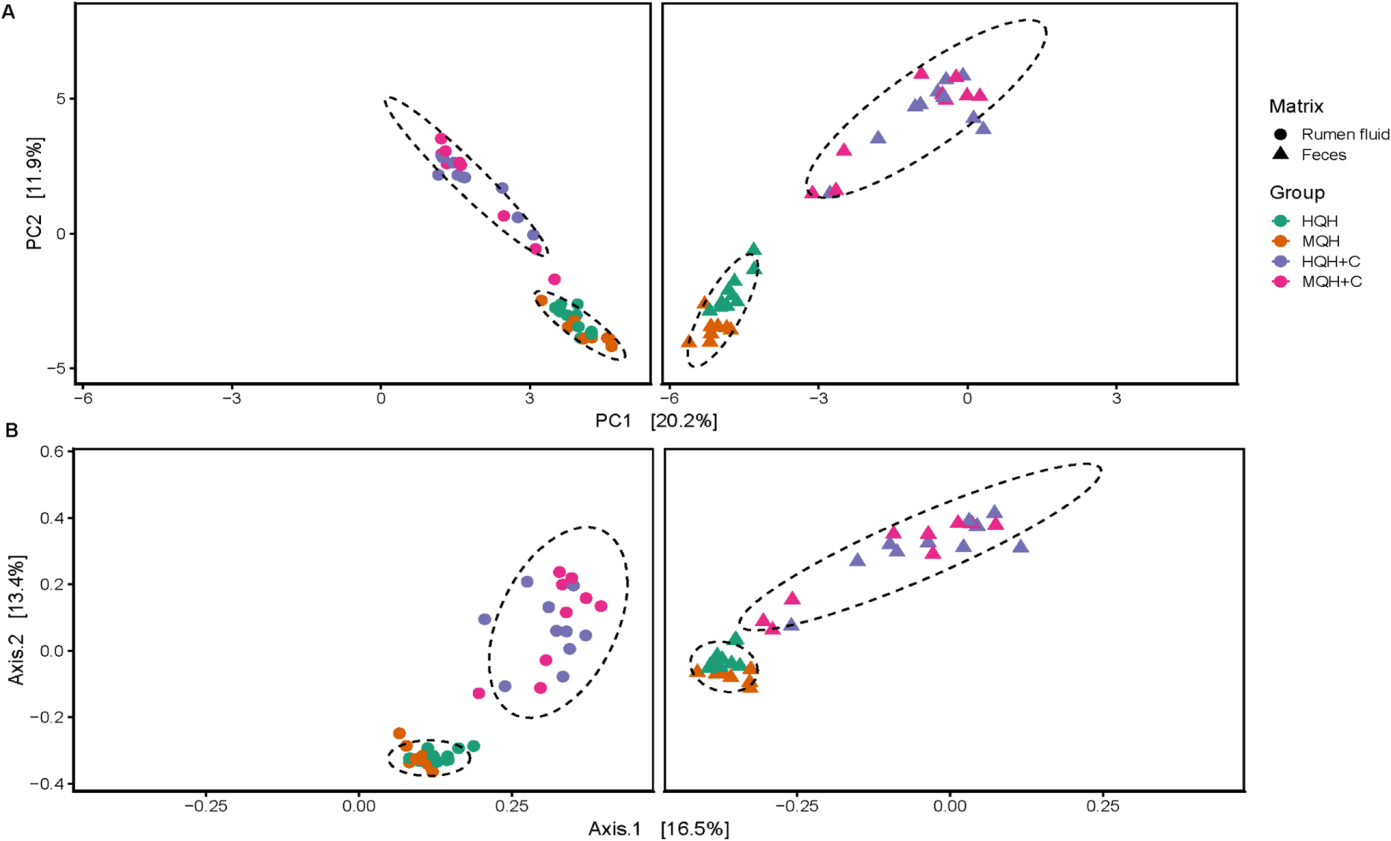
Principal Coordinates Analysis plots in ruminal fluid and feces based on Aitchison (**A**) and Bray–Curtis distances (**B**).

Beta-diversity showed a significant effect for dietary group (R^2^ = 0.08–0.1, *P* = 0.001) in both Aitchison and Bray–Curtis distances (Fig. 2A,B). However, when testing for the separate effect of hay quality and concentrate, no significant effect was found regarding hay quality (*P* > 0.1), but for concentrate (R^2^ = 0.1–0.12, *P* = 0.001). Therefore, further analysis was conducted based on these two categories, i.e. hay quality and concentrate inclusion. Inclusion of concentrate decreased Shannon, Simpson and Fisher’s alpha diversity index, as well as number of ASVs in the ruminal fluid (*P* < 0.01; Table 1). Likewise, when compared to medium-quality hay, feeding high-quality hay decreased microbial richness and diversity (*P* ≤ 0.05). No interaction between hay quality and concentrate was found. However, both Simpson and Fisher’s alpha diversity index tended to be lower in HQH + C than in the other treatments (each *P* = 0.08).

**Table 1:** Comparison of alpha-diversity estimates between treatment groups in ruminal fluid and feces on d 91.

	Groups				SEM^a^	*P* value		
	MQH	HQH	MQH + C	HQH + C		Hay	Concentrate	Hay × Concentrate
**Ruminal fluid**								
Observed ASVs	1006	800	361	334	56	0.04	< 0.01	0.11
Shannon	4.98	4.75	3.47	3.22	0.12	0.05	< 0.01	0.93
Simpson	0.98	0.98	0.92	0.88	0.009	0.05	< 0.01	0.08
Fisher’s alpha	167	129	53.1	46.7	9.06	0.02	< 0.01	0.08
**Feces**								
Observed ASVs	1208^b^	836^c^	428^d^	384^d^	46	< 0.01	< 0.01	< 0.01
Shannon	5.65	5.26	4.36	4.23	0.12	0.03	< 0.01	0.27
Simpson	0.99	0.98	0.97	0.96	0.005	0.55	< 0.01	0.63
Fisher’s alpha	218^b^	153^c^	66.4^d^	57.3^d^	7.38	< 0.01	< 0.01	< 0.01

Superscript lowercase letters b to d indicate difference (P < 0.05).

*MQH* medium-quality hay without concentrate, *HQH* high-quality hay without concentrate, *MQH* + *C* medium-quality hay with 70% concentrate, *HQH* + *C* High-quality hay with 70% concentrate.

^a^Standard error of the mean.

In feces, observed ASVs (*P* < 0.01) and Fisher’s alpha diversity index (*P* < 0.01) were lowest in MHQ + C and HQH + C, intermediate in HQH and highest in MQH, therefore differentiating between hay qualities only without concentrate supplementation (Table 1). Moreover, similar patterns as found in ruminal fluid were also present for the main effect of concentrate inclusion in feces, i.e. reduced alpha diversity measures and less observed ASVs (each *P* > 0.01). Similarly, compared to MQH, HQH feeding resulted in less ASVs (*P* < 0.01) and lower estimates of Shannon (*P* = 0.03) and Fisher indices (*P* < 0.01).

**Figure 3:**
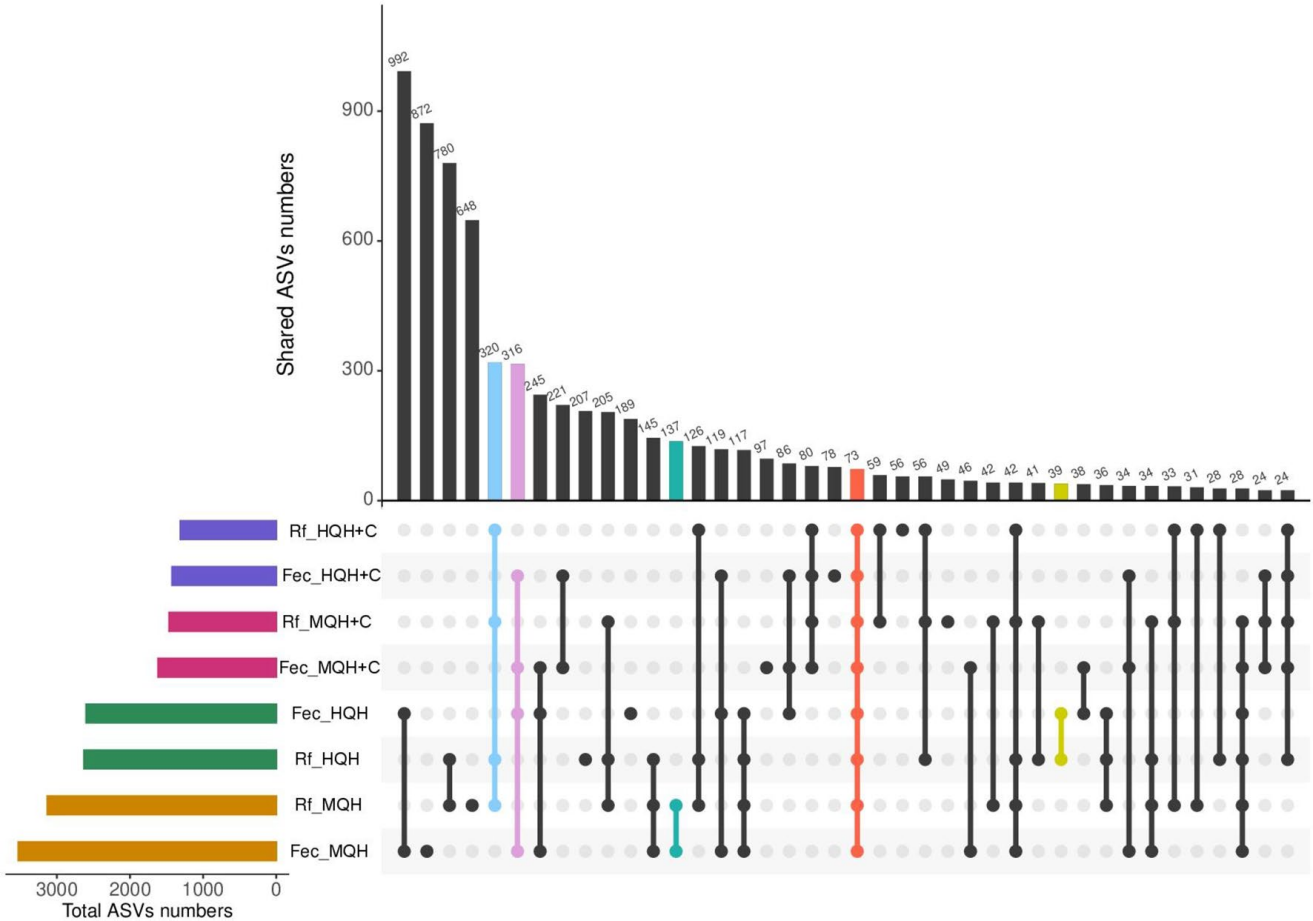
Numbers of shared ASVs for solid feeding groups, separated by ruminal fluid and feces on d 91.

As illustrated in Fig. 3, 73 ASVs were present for all ruminal fluid and feces samples, whereas 320 ASVs were shared between all ruminal fluid samples and 316 ASVs between all fecal samples. Regarding the specific solid diets, 137 and 39 ASVs were exclusively found in both ruminal fluid and feces for MQH and HQH, respectively. No shared ASVs between feces and rumen fluid were found for HQH + C and MQH + C.

### Between-group differences among microbial genera in ruminal fluid and feces

**Figure 4:**
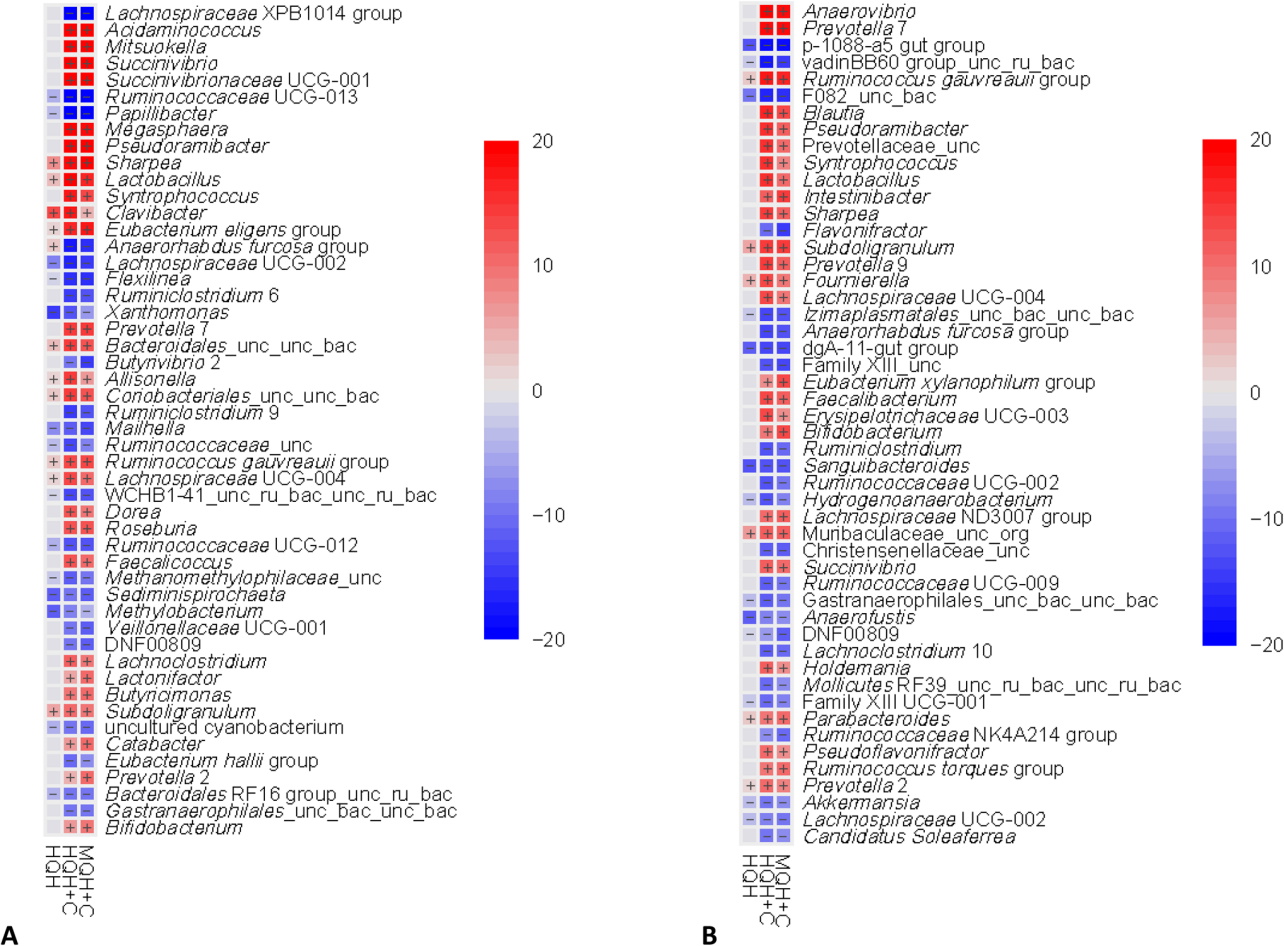
(**A**,**B**) The top 50 bacterial genera with significant associations (-log(Q)*coefficient) to dietary treatment groups in ruminal fluid and feces on d 91.

Differences between MQH, HQH, MQH + C and HQH + C were inspected at the microbial genus level using MQH as reference. The top 50 genera with significant associations in ruminal fluid and feces is shown in Fig. 4A,B, respectively.

In ruminal fluid, *Sharpea*, *Shuttleworthia*, *Subdoligranulum* and *Clavibacter* significantly increased in HQH, when compared with MQH (Supplementary Table 4; coefficient > 2.00, Q < 0.05). *Rhizobium*, *Anaeroplasma*, an uncultured rumen RF16 group bacterium, *Elusimicrobium*, *Oscillospira* and *Xanthomonas* decreased (coefficient < − 2.00, Q < 0.05). In the HQH + C and MQH + C groups, 79 and 75 genera were significantly affected when compared with the MQH group, respectively. *Succinivibrionaceae* UCG-001, *Sharpea*, *Prevotella* 7, *Succinivibrio*, *Acidaminococcus*, *Megasphaera*, *Mitsuokella* and *Lactobacillus* were the genera that most increased in MQH + C and HQH + C (coefficient > 5.90, Q < 0.05). *Ruminococcus* 1 and *Fibrobacter* decreased in MQH + C (coefficient < − 2.00, Q < 0.05), but were not affected in the HQH + C group. *Lachnospiraceae* XPB1014 group, *Ruminiclostridium* 9 and two uncultured rumen bacteria were among those that most decreased in HQH + C group (coefficient < − 4.00, Q < 0.05). The same two uncultured microbes were also amongst the microbial genera that mostly decreased in MQH + C, together with *Saccharofermentans*, *Ruminiclostridium* 9, *Butyrivibrio* 2 and *Lachnospiraceae* XPB1014 group (coefficient < − 4.00, Q < 0.05).

In feces, a total of 17 bacterial genera were significantly different between MQH and HQH, while 92 and 81 changed between MQH and HQH + C and MQH and MQH + C, respectively (coefficient > ± 2.00, Q < 0.05, Supplementary Table 4). *Prevotellaceae* NK3B31 group, *Muribaculaceae* uncultured organism, *Candidatus Stoquefichus*, *Alloprevotella*, *Subdoligranulum*, *Angelakisella*, *Muribaculum* and *Sutterella* were the genera that mostly increased in HQH when compared with MQH (coefficient > 2.00, Q < 0.05), while *Akkermansia*, *Methanocorpusculum*, *Anaerosporobacter*, p-1088-a5 gut group, dgA-11 gut group and 4 other unclassified genera decreased (coefficient > 2.00, Q < 0.05). Differences between MQH and MQH + C and HQH + C were quite similar in both concentrate groups. In HQH + C, 44 genera were found to increase when compared with MQH (coefficient > 2.00, Q < 0.05). From these, 36 were also found to increase in MQH + C. *Collinsella*, *Agathobacter*, *Clostridium *sensu stricto 1, *Kandleria*, *Faecalicoccus* and CAG-56 were the genera which differed between MQH and HQH + C, but not between MQH and MQH + C. Other 48 bacterial genera were found to significantly decrease in HQH + C (coefficient > 2.00, Q < 0.05), while 44 decreased in MQH + C (coefficient < − 2.00, Q < 0.05). In comparison with MQH, *Streptococcus* was the only genera found to decrease in MQH + C (coefficient = − 2.18, Q < 0.02), but did not change in HQH + C. In contrast, *Eubacterium hallii* group, *Eubacterium nodatum* group, *Alistipes*, *Campylobacter* and an *Izimaplasmatales* gut metagenome bacteria significantly decreased in HQH + C, but not in MQH + C group compared to MQH (coefficient > − 2.0, Q < 0.05).

### Cluster separation of fecal enterotypes

**Figure 5:**
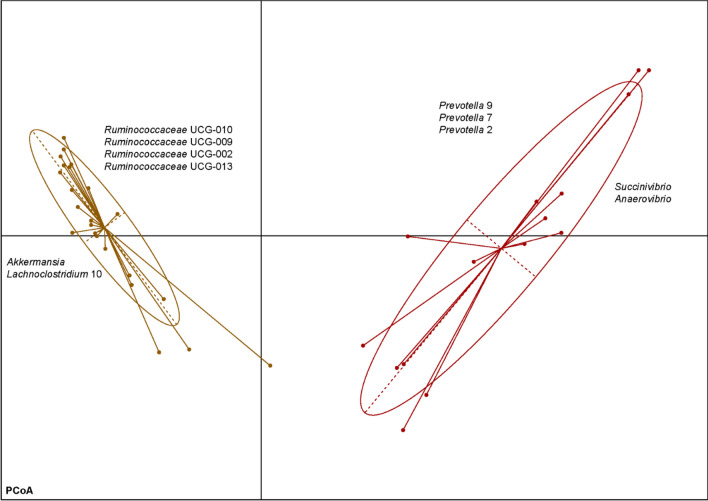
Principal Coordinates Analysis plot for Partitioning Around Medoids-based enterotyping in feces on d 91. Cluster 1 is based on 23 animals with 19 fed no concentrate and 4 fed 70% concentrate and cluster 2 is based on 15 animals with all being fed 70% concentrate.

**Figure 6:**
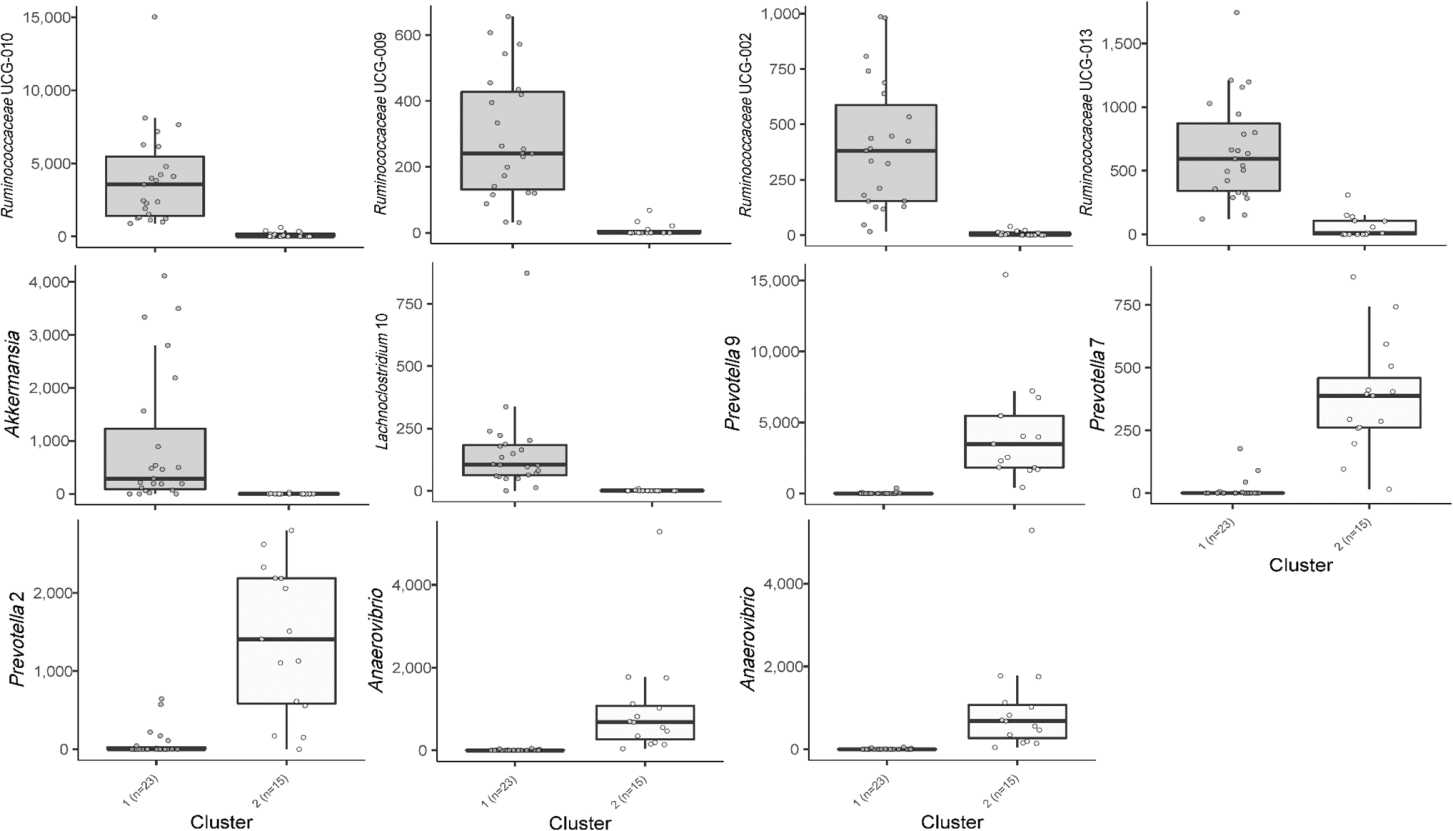
Prevalent bacterial genera in the two fecal enterotypes on d 91. Cluster 1 is based on 23 animals with 19 fed no concentrate and 4 fed 70% concentrate and cluster 2 is based on 15 animals with all being fed 70% concentrate.

Partitioning Around Medoids (PAM)-based clustering revealed the presence of two optimal clusters (Supplementary Fig. 3, Fig. 5: cluster 1, n = 23 calves; cluster 2, n = 15 calves). Cluster 1 was particularly enriched in *Ruminococcaceae* genera UCG-010, UCG-009, UCG-002, UCG-013, *Akkermansia* and *Lachnoclostridium* 1 (Fig. 6), being dominated by samples from animals fed no concentrate in their diets (19 animals with no concentrate and 4 animals fed 70% concentrate). The second enterotype was prevalent among animals in groups fed 70% concentrate (15 animals fed 70% concentrate), and has an enrichment of genera *Prevotella* 9, 7, 2, *Succinivibrio* and *Anaerovibrio* (Fig. 6).

## Discussion

Our study aimed to investigate changes in the microbiota compositions in ruminal fluid and feces of calves between milk feeding on d 7 and after being weaned consuming a solid diet differing in concentrate inclusion and hay quality on d 91. Our companion papers describe differences in growth performance as well as ruminal and fecal fermentation patterns due to these dietary factors^8,9^. Thus, we explored the impact of these different carbohydrate sources in the gut sections with the by far highest microbial activity, i.e. rumen and hindgut, and anticipated finding distinct microbial communities between the feeding groups in both matrices, such as more fibrolytic bacteria with pure hay feeding.

The present results showed a clear change in the bacterial community composition in both ruminal fluid and feces when calves were transitioned from acidified milk to a solid diet, which in turn was affected by hay quality and particularly concentrate supplementation. Regarding the overall changes from early life to post-weaning, we observed an increased microbiota complexity, i.e. richness and diversity, from d 7 to d 91 as evidenced by increasing alpha diversity metrics in both matrices, suggesting a successful colonization and establishment of the rumen and hindgut and therefore the dominance of a microbial fermentation-based digestion. Interestingly, although calves were functionally monogastrics on d 7, the ruminal fluid harbored more than twice as much ASVs as feces. At first sight, this observation was rather unexpected since the esophageal groove closure passes milk directly into the abomasum^1^, therefore minimizing fermentative substrate availability in the rumen. Accordingly, total SCFA concentrations were around fourfold lower in ruminal fluid than in feces on d 7, i.e. 15.9 µmol/g and 68.9 µmol/g, respectively^8^. However, our diversity data matches with other studies that observed comparable variation of bacterial species, including potent fiber-degrading members, in the rumen of milk-fed calves within their first week of life^4,10,11^. On d 91, ASV numbers were generally higher compared to d 7 but no longer different between the two gut segments, thus indicating a catch-up effect regarding subsequent maturation and specialization of the hindgut communities. This may be explained by an increased variety of fermentable substrates entering the distal gut section with solid feeding as is also evidenced by the higher fecal SCFA concentrations found on d 91 when compared to d 7^8^.

On a compositional level, major shifts in predominant bacterial genera occurred during the transition to solid feed as exemplified by the sharp decrease of *Bifidobacterium* in feces, which is typically present with milk feeding but diminishes after weaning^12^; as well as the increasing abundances of fiber degradation-associated genera, such as *Lachnospiraceae* UCG-010 and *Rikenellaceae* RC9 gut group^13,14^. Likewise, major fibrolytic genera were among those that increased most in ruminal fluid between d 7 and d 91, i.e. *Prevotellaceae* YAB2003 group, *Lachnospiraceae* NK3A20 group and *Pseudobutyrivibrio*^7,13,14^—the latter one has also been found to increase during pure HQH feeding to adult Holstein cows, likely not only due to cellulolytic activity, but as well its efficient utilization of fructans and water-soluble carbohydrates^7^, which were particularly high in the HQH diet^9^. Moreover, all of these three bacterial genera were also strongly positively correlated with ruminal acetate as well as butyrate concentration, thus, their proliferation may explain the elevated butyrate proportions found in the rumen of HQH fed calves^8^.

Despite the described general increase in diversity due to solid feed introduction, both dietary factors, i.e. inclusion of concentrate and hay quality, determined the nature of the shifts in the ruminal as well as fecal microbiota. In ruminal fluid and feces, concentrate inclusion drastically reduced bacterial diversity and richness, which may be explained by the fact that a large part of the diet consisted of rapidly fermentable carbohydrates, i.e. sugars and starch that are efficiently utilized by a certain proportion of the microbiota, which therefore outcompete other microbial members. A direct pH-mediated suppression of fibrolytic bacteria^15^ may be excluded as measured ruminal and fecal pH values maintained physiologically in all groups—though it has to be considered that continuous pH recording was not possible, but was determined weekly in morning samples^8^. Shifts towards a grain feeding-dominated microbiota were also present at genus level in the rumen with higher abundances of *Lactobacillus* and *Megasphaera* at the expense of genera belonging to fibrolysis-associated *Ruminococcaceae* and *Lachnospiraceae*. Likewise, more lactobacilli but less *Ruminococcaceae* and *Lachnospiraceae* in feces of calves receiving concentrate confirmed our hypothesis. In case of *Lactobacillus*, this also matches observations made in concentrate supplemented vs. non-supplemented beef calves at 92 d of age, which however were not completely weaned^5^. Within dietary treatment groups, *Ruminococcus* 1 and *Fibrobacter* were more abundant in the ruminal fluid of animals from the MQH group when compared to MQH + C. However, this was not true in comparison with HQH + C, which might indicate the potential of HQH to better maintain a cellulolytic community in the rumen than MQH when concentrate is included. Additionally, since calves in the MQH + C group tended to show a stronger sorting against hay than HQH + C, the proportion of hay was actually higher in the HQH + C group^9^.

Regarding the impact of hay quality, MQH and HQH each led to the development of a specific bacterial subset along the entire gastrointestinal tract as evidenced by the 137 and 39 ASVs being exclusively present in both gut segments for MQH and HQH, respectively. Thus, our hypothesis of the development of bacterial communities in the intestine to be also driven by hay quality seems to be supported. Interestingly, no specific ASV profiles were observed when concentrate was included in the solid diet, i.e. neither for MQH + C nor for HQH + C, which was further shown by similar ASV numbers and Fisher’s alpha metric in feces. Thus, concentrate inclusion seemed to superimpose the differences between hay qualities.

As found for concentrate inclusion, feeding of HQH as well led to reduced bacterial diversity compared to MQH in both gut segments—however, to a much lesser extent. Likewise, also fewer changes in bacterial abundances in response to MQH or HQH were observed at genus level. Still, the explanation for the effect of hay quality might be analogous to that described for concentrate, i.e. bacteria that rapidly utilize the sugars of HQH suppress the proliferation of others, which consequently reduced diversity. However, concentrate addition altered the bacterial profile in the first place and was mainly responsible for changes in microbial composition and decreased bacterial diversity, which is in line with observations made in high-grain fed dairy cows^16,17^. It is noteworthy that Klevenhusen et al.^7^ found hay quality to be a coequal driver for differences in rumen microbiota structure of dry cows as concentrate inclusion—although highest concentrate addition in their study amounted for 40% in DM, which thus suggests that amount of concentrate could be decisive for the extent of its influence. Also, adult cows are fully developed ruminants and probably less susceptible to long-lasting microbiome interventions compared to calves^18,19^. Furthermore, our clustering analysis revealed the presence of two enterotypes that were differentiated by concentrate inclusion but not hay quality, thus again underlining the essential role of concentrate as an impact factor on the development of a specialized gut microbiota. Without concentrate, fibrolytic bacteria of *Ruminococcaceae* groups were predominant, whereas several groups of *Prevotella* characterized the concentrate-associated enterotype, a genus that is known to typically proliferate with concentrate feeding of ruminants^20,21^.

Feeding of solely HQH enabled a similar feed intake and growth performance as found for MQH + C^9^, meaning economic benefits in calf rearing, as well as improved rumination activity and rumen fermentation pattern^8^. Similarly, our results revealed a more diverse bacterial composition in ruminal fluid and feces with this HQH feeding regime and thus suggested a more stable gut microbiome that may imply that those calves are potentially more disease-resistant^22^. In a long-term perspective, the better understanding on how concentrate inclusion and hay quality influence the calves’ gut microbiota may allow its beneficial manipulation during early-life and by this support calves to meet the future demands of modern dairy production.

## Conclusion

The transition of the milk-fed calf to a ruminant with an almost fully developed forestomach system was expressed by higher diversity and changes in bacterial composition in both ruminal fluid and feces. Thereby, the contrasts in carbohydrate sources clearly affected the manifestation of the microbial structure, which was predominantly driven by concentrate inclusion, but to a lesser extent by hay quality, as well. Feeding concentrate generally decreased bacterial diversity, promoted the abundance of potential starch degraders and reduced the presence of key genera associated with fiber degradation in both gut locations. Likewise, two fecal enterotypes separated by concentrate inclusion were found, whereas MQH and HQH both led to the evolvement of specific ASV profiles in the calves’ gut.

## Methods

### Animals

The methods and protocols in this experiment followed the ARRIVE guidelines and were approved by the Austrian national authority according to the law for animal experiments Tierversuchsgesetz 2012-TVG (GZ: BMBWF-66.019/0016-V/3b/2019). Four cohorts of 10 Holstein–Friesian dairy calves (5 males and 5 females per group; total n = 40) were kept at the Agricultural Research and Education Centre (AREC) Raumberg-Gumpenstein (47° 30′ N, 14° 6′ E), Austria.

### Diets and experimental design

Detailed information about animals, feeding, feedstuffs, experimental design, systemic health variables and gut fermentation are given in our companion papers^8,9^. Briefly, all calves were kept in individual boxes and fed acidified milk according to a standard milk feeding plan until they were weaned on d 84 (i.e. first 28 d ad libitum milk feeding following a stepwise weaning program until d 84). From day of birth onward, calves had also ad libitum access to solid feed and were allocated to 2 × 2 treatment design with two dietary factors (hay quality and concentrate inclusion) of two levels each (hay of either medium or high quality and without or with the inclusion of concentrate). Therefore, the following four experimental diets were fed to calves (n = 10/group) ad libitum: (1) 100% medium-quality hay (MQH), (2) 100% high-quality hay (HQH), (3) 30% medium-quality hay with 70% concentrate (MQH + C), or (4) 30% high-quality hay with 70% concentrate (HQH + C). Details on the nutritional composition of milk and solid feedstuffs are presented in detail in Terler et al.^9^ and summarized in Supplementary Table 5.

### Rumen and fecal sampling

Samples of ruminal fluid and feces were collected at d 7 (body weight 46.5 ± 2.5 kg; mean ± standard deviation) and at d 91 (body weight 113.2 ± 6.9 kg) at 0900 h. To collect ruminal fluid, a manually operated vacuum pump was used. The tube was gently placed in the mouth of the animal until reaching the rumen. The first 10 ml were discarded to minimize saliva contamination and approximately 30 ml were recovered and filtered through gauze compresses (Wilhelm Weisweiler GmbH & Co. KG, Münster, Germany). Aliquots were stored in 8 ml Eppendorf tubes at − 80 °C until analysis. Fecal samples were obtained rectally using a new palpation sleeve for each collection and subsamples were stored in 2 ml Eppendorf tubes at − 80 °C until analysis.

### DNA extraction and sequencing

Isolation and purification of microbial DNA was performed using the DNeasy PowerSoil Kit (Qiagen, Hilden, Germany) with minor modifications according to Hartinger et al.^23^. Briefly, after fluid samples were thawed on ice, 800 μl of each sample was transferred to a bead beating tube provided in the kit. In the case of feces, 250 mg input material were used. After adding of 60 µl C1 to each sample, all samples were incubated at 95 °C for 5 min. Following a centrifugation at 10,000*g* for 2 min, supernatants were collected in fresh tubes and placed on ice for later procession. Lysozyme (100 µl of 100 mg/ml, Sigma-Aldrich, Vienna, Austria) and mutanolysin (10 µl of 2.5 U/ml, Sigma-Aldrich, Vienna, Austria) were added to each pellet and incubated at 37 °C for 30 min. Subsequently, 21 µl of 19 mg/ml proteinase K (Sigma-Aldrich, Vienna, Austria) was added to each pellet and incubated at 37 °C for 1 h, followed by mechanical disruption using a homogenizer (FastPrep-24, MP Biomedical, Santa Ana, CA, USA). After centrifugation, the supernatant of each sample was collected and added to the previously separated supernatant. Protein degradation, removal of PCR inhibitors and cell debris were performed by using the provided buffers C2–C5 and subsequent centrifugation steps. Finally, supernatants were loaded on silica-gel membranes and total genomic DNA was eluted in 100 µl of C6 buffer. Total DNA was measured on a Qubit Fluorometer 4.0 (Life Technologies, Carlsbad, CA, USA) using the Qubit dsDNA HS Assay Kit (Life Technologies, Carlsbad, CA, USA). Amplicon sequencing was performed using Illumina MiSeq paired-ends sequencing technology (Microsynth AG, Balgach, Switzland). Targeted amplification of the hypervariable region V4 of bacterial 16S rRNA gene (2 × 250 bp) was performed using the primers 515F (5′-GTGCCAGCMGCCGCGGTAA-3′) and 806R (5′-GGACTACHVGGGTWTCTAAT-3′) from Caporaso et al.^24^, as V4 has a lower error rate than other hypervariable regions^25^ and was recently found to be superior to V3–V5 in terms of capturing the microbial diversity in the rumen^26^. Multiplexed libraries were constructed by ligating sequencing adapters and indices onto purified PCR products using the Nextera XT Sample Preparation Kit (Illumina, Balgach, Switzerland). Primers were trimmed and corresponding overlapping paired-end reads were stitched by Microsynth (Microsynth AG, Balgach, Switzland).

### Bioinformatics and statistical analyses

Merged reads were processed using the software package Quantitative Insights into Microbial Ecology (QIIME2 v2020.2^27^;). Read quality was inspected using FASTQC v. 0.11.5^5^ and sequence data was quality filtered using the *q-score-joined* plugin with a minimum acceptable PHRED score of 20. Denoising into amplicon sequence variants (ASVs) was obtained using Deblur^28^ by trimming all reads to a length of 250 nucleotides and removing low abundance features. Representative sequences and feature tables were filtered to exclude all features classified as mitochondria or chloroplast. All resulting filtered ASVs were aligned with mafft^29^ and used to construct a phylogeny with fasttree2^30^. Taxonomy was assigned to ASVs using a classify‐sklearn naïve Bayes taxonomy classifier trained with the 515F/806R primer set against the SILVA 132 99% OTUs reference sequences. Rooted tree, taxonomy and filtered feature table were used as an input to phyloseq in R. Statistical analysis of microbial alpha-diversity was performed using PROC UNIVARIATE to test for normality of the data followed by PROC MIXED in SAS (v. 9.4, SAS Institute Inc., Cary, NC, USA) with matrix, hay quality, concentrate feeding as fixed effects and animal and sex as random effects. Significance was declared at *P* ≤ 0.05 and trends were considered at 0.05 < *P* ≤ 0.10. LSMEANS were compared with the PDIFF option using the Tukey post-hoc test. Differences in beta-diversity matrices were calculated with the vegan package using the *adonis2* function. Enterotyping based on Partitioning Around Medoids clustering using the Jensen–Shannon divergence was conducted on the relative abundance matrix of microbial genera^31–33^. The optimal number of clusters was determined by the Calinski–Harabasz Index^34^. Differential abundance of microbial genera was done in MaAsLin2^35^. Spearman correlations have been calculated based on a subset of SCFA data^8^ using Hmisc v 4.6.0. Only correlations with r > 0.7 or < − 0.7 and *P* value < 0.05 were considered.

## Supplementary Information

Supplementary Legends.

Supplementary Table 1.

Supplementary Table 2.

Supplementary Table 3.

Supplementary Table 4.

Supplementary Table 5.

Supplementary Figures.

## Data Availability

Sequences have been submitted to the National Center for Biotechnology Information (NCBI) sequence read archive (SRA) under the accession number PRJNA818123.
