# Supplementary material for: Shift of dietary carbohydrate source from milk to various solid feeds reshapes the rumen and fecal microbiome in calves
Source: Sci Rep. 2022 Jul 20;12:12383. doi: 10.1038/s41598-022-16052-2 (PMC9300698; doi:10.1038/s41598-022-16052-2)
Supplement: Supplementary file 1 — Supplementary Legends. [file 41598_2022_16052_MOESM1_ESM.docx]

**Supplementary data captions**

**Supplementary Table 1.** Taxa at phylum, family and genus level, given as total and as % of read counts in rumen fluid and feces.

**Supplementary Figure 1.** Top 25 most abundant genera in rumen fluid and feces.

**Supplementary Table 2.** Microbial genera found at d 7.

**Supplementary Table 3.** Differential abundance between d7 and d91.

**Supplementary Table 4.** Between-group differences among microbial genera in ruminal fluid and feces.

**Supplementary Table 5.** Chemical composition of milk and solid feedstuffs.

**Supplementary Figure 1.** Divergent composition at genus level between d7 and d91 in ruminal fluid and feces.

**Supplementary Figure 2.** Correlation network between pH, SCFA and bacterial genera in ruminal fluid on d 91. Negative correlations are in blue and positive correlations in red.

**Supplementary Figure 3.** Optimal number of clusters based on Partitioning Around Medoids (PAM)-based clustering.
