# Supplementary material for: Shift of dietary carbohydrate source from milk to various solid feeds reshapes the rumen and fecal microbiome in calves
Source: Sci Rep. 2022 Jul 20;12:12383. doi: 10.1038/s41598-022-16052-2 (PMC9300698; doi:10.1038/s41598-022-16052-2)
Supplement: Supplementary file 6 — Supplementary Table 5. [file 41598_2022_16052_MOESM6_ESM.pdf]

Supplementary Table 5.

Item	Milk	Medium quality hay	High quality hay	Concentrate
Dry matter (g/kg)	130 ± 2	899 ± 24	877 ± 30	891 ± 13
Crude protein (g/kg DM)	260 ± 3	149 ± 29	210 ± 11	193 ± 9
Ether extract (g/kg DM)	322 ± 6	18 ± 3	24 ± 3	18 ± 2
Ash (g/kg DM)	58 ± 1	76 ± 7	86 ± 3	39 ± 11
NDF[1] (g/kg DM)	-	522 ± 24	455 ± 15	204 ± 12
ADF[2] (g/kg DM)	-	329 ± 15	247 ± 11	66 ± 5
ADL[3] (g/kg DM)	-	49 ± 7	23 ± 3	13 ± 2
NFC[4] (g/kg DM)	360 ± 6	235 ± 34	225 ± 16	547 ± 16
WSC[5] (g/kg DM)	-	124 ± 34	205 ± 10	-
ME[6] (MJ/kg DM)	19.2 ± 0.1	9.4 ± 0.4	11.2 ± 0.2	13.5 ± 0.2
$peNDF_{>8mm}$[7] (% DM)	-	38.0 ± 6.3	43.1 ± 0.5	-

[1]NDF = Neutral detergent fiber; [2]ADF = Acid detergent fiber; [3]ADL = Acid detergent lignin; [4]NFC = (1,000 – ash – crude protein – ether extract – NDF); [5]WSC = Water-soluble carbohydrates; [6]ME = Metabolizable energy; [7]peNDF>8mm = physically effective NDF >8 mm
