# Supplementary figures and images for: Shift of dietary carbohydrate source from milk to various solid feeds reshapes the rumen and fecal microbiome in calves
Source: Sci Rep. 2022 Jul 20;12:12383. doi: 10.1038/s41598-022-16052-2 (PMC9300698; doi:10.1038/s41598-022-16052-2)

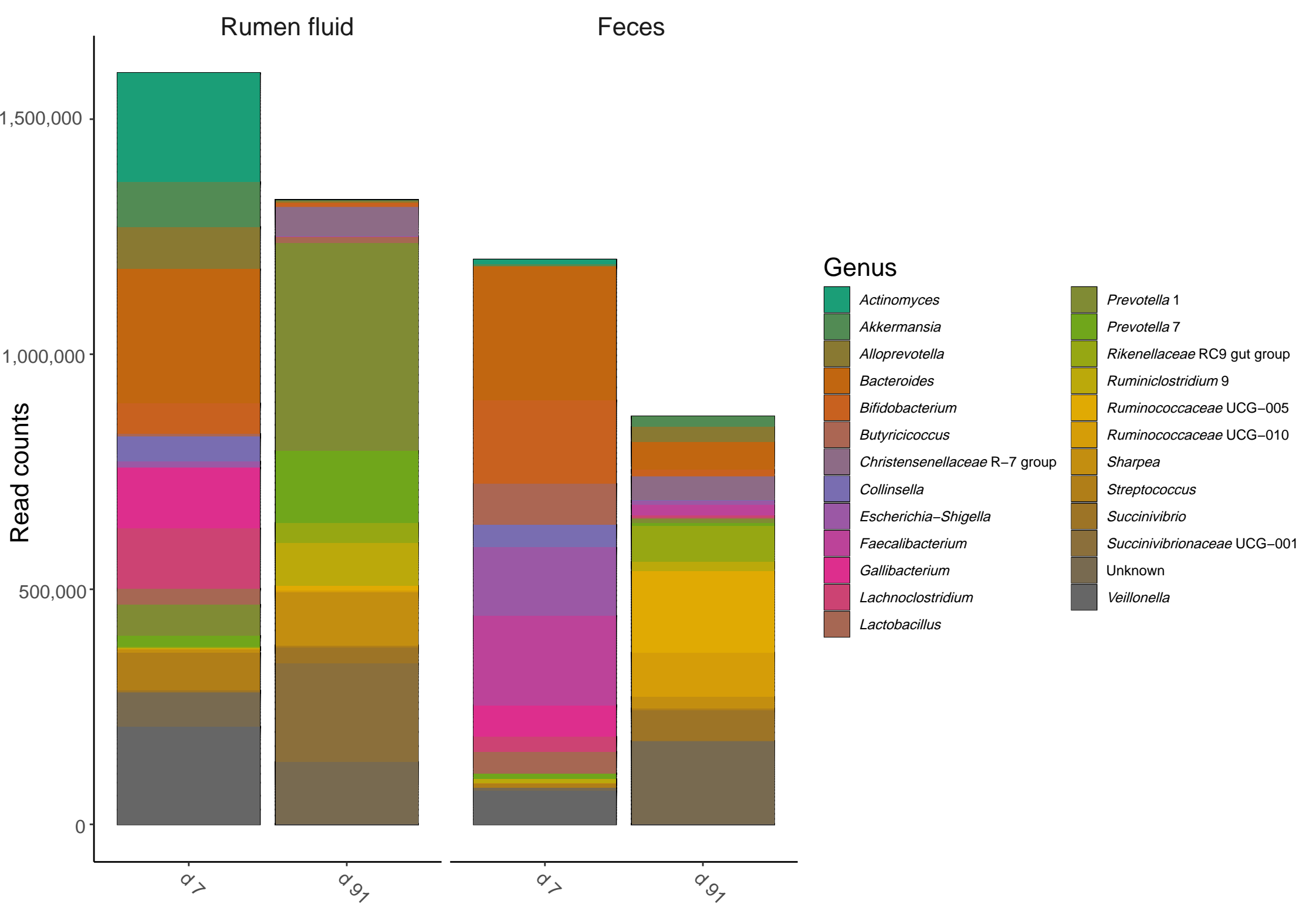

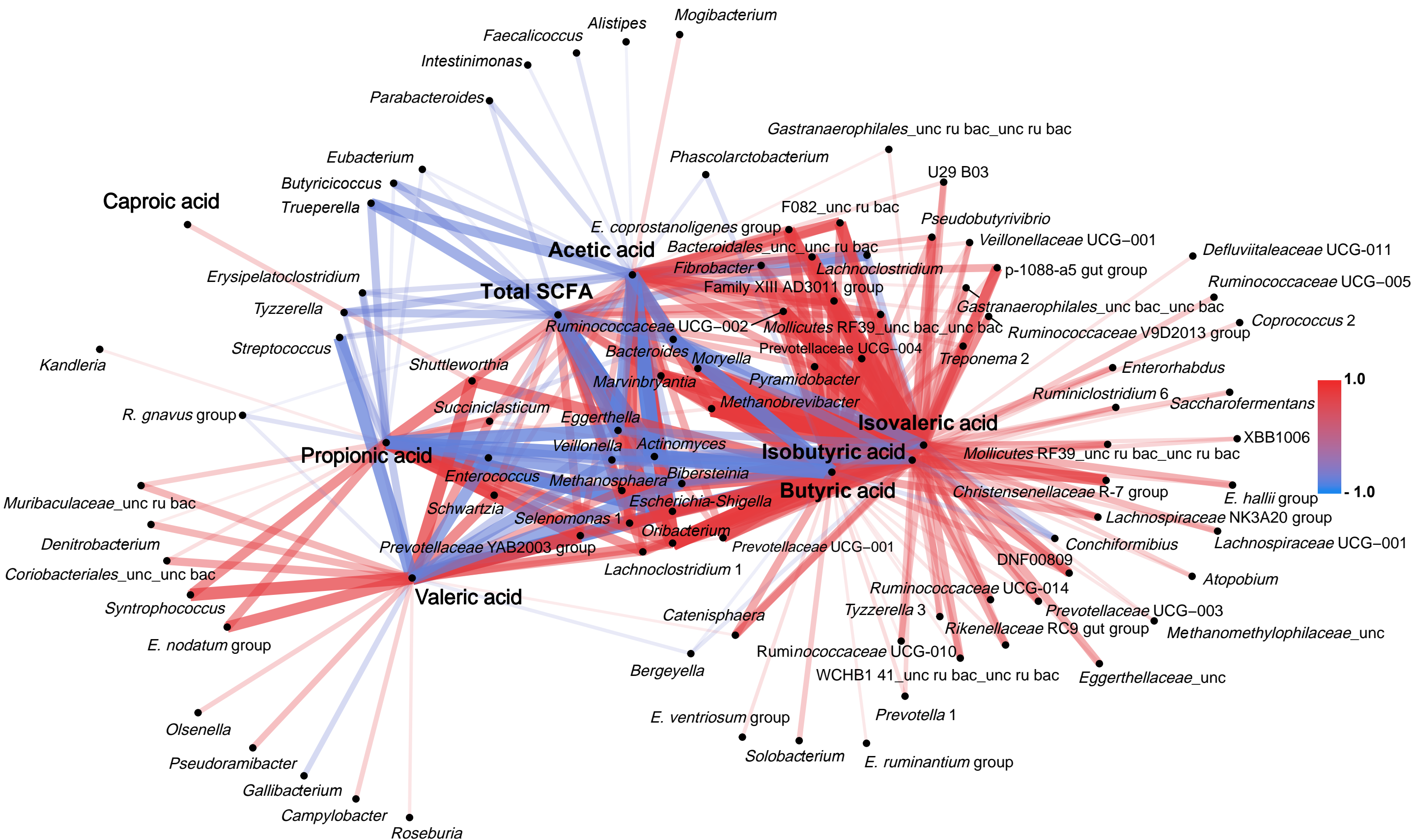

**Optimal number of clusters**

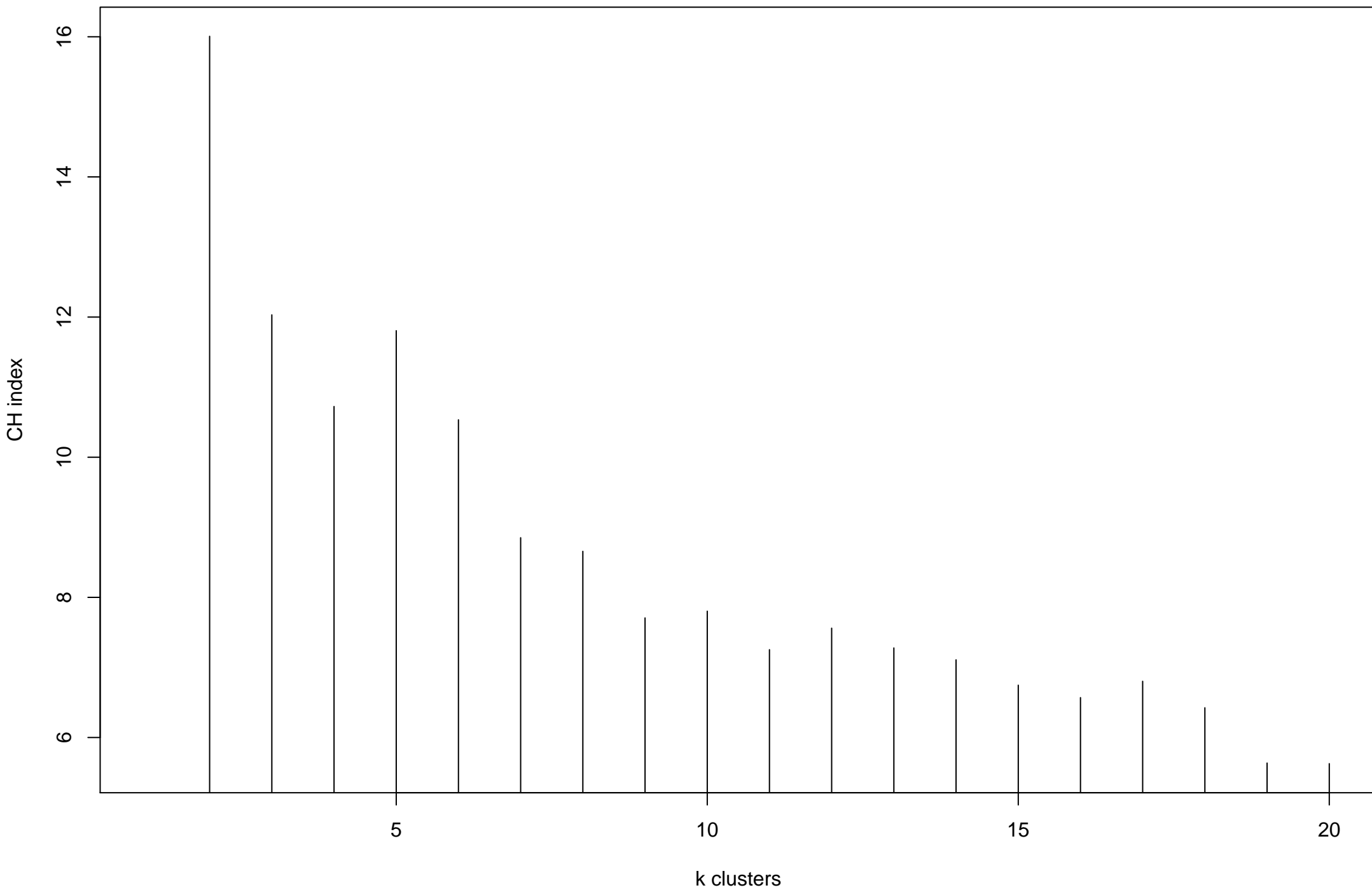

Supplement: Supplementary file 7 — Supplementary Figures. [file 41598_2022_16052_MOESM7_ESM.pdf]
